# Analysis of the Innovative Channel Strut Concept Manufactured by Roll-Forming

**DOI:** 10.3390/ma15031107

**Published:** 2022-01-31

**Authors:** Andrzej Kochański, Piotr Czyżewski, Robert Cacko, Mariusz Roznowski

**Affiliations:** 1 Faculty of Mechanical and Industrial Engineering, Warsaw University of Technology, 02-524 Warszawa, Poland; andrzej.kochanski@pw.edu.pl (A.K.); piotr.czyzewski@pw.edu.pl (P.C.); 2 MFO S.A., 96-500 Sochaczew, Poland; m.roznowski@mfo.pl

**Keywords:** steel console, steel profile, FEM analysis, roll-forming, load-bearing systems

## Abstract

Due to the wide use of channel strut components, manufacturing is implemented in many industrial plants. Standard technology of profiles is based on welding of two parts of the profile and requires the regalvanizing of the joint zone causes. Thus, the production is challenging to automate on a single line. The main idea of the article is to present a concept of a channel strut, a cold-formed continuous metal component with an open or closed profile section. It would serve as a cantilever support instead of a standard solution. In the article, a unique lock system combination is proposed and analyzed both numerically and experimentally to provide steadiness of the strut without welding or other joining techniques. Two main lock shapes—semicircular and triangle—were proposed with some variations in the cutting plane. Analyses were carried out for three main profile cross-sections with different dimensions, based on the current industrial applications. The semicircular type of the lock was found to be the most stable, giving optimal strength to the strut under assumed loading, comparable to traditional solutions. The commercial FEM software MSC Marc was used for the numerical analysis.

## 1. Introduction

The first generation of modern roll-forming machines using rotating rollers instead of stationary dies was built commercially in 1920 [1]. Initially, they were used to form blanks that had been sheared from sheet metal. With the development of sheet material in the form of tape wrapped in rolls/coils, roll-forming improved into the process known today: a continuous process of production of profiles production on a line based on unique stations equipped with rollers [1]. Roll-forming is often classified as an incremental forming method because it consists of gradually and continuously bending a steel profile. The procedure begins with the unrolling the strip of a steel coil, which usually corresponds to the development of the finished profile. The strip is then flattened in a succession of several rolls, of which the shapes and numbers are variable, depending on the complexity of the desired steel profile cross-section [1,2]. Usually, it is associated with other metal forming and metalworking technologies, such as stamping, cutting, machining, welding, etc., for a final product shape.

Components commonly manufactured from roll-formed profiles are struts and consoles. These constructions are mainly used as load-bearing elements in electrical, gas, or ventilation installations/systems. They are mounted on the walls or ceilings as room-support pipelines, cable trays, or ventilation ducts. The method of assembly and load causes the consoles to be subjected to bending or stretching forces. Brackets can act as an individual support/strengthener and work in the console team [2]. In addition, they can be one of the elements of more complex structures with a complex load condition. Industrial applications include racks and shelving, partitions, production line supports, trolley systems, wall framing, etc.

**Figure 1:**
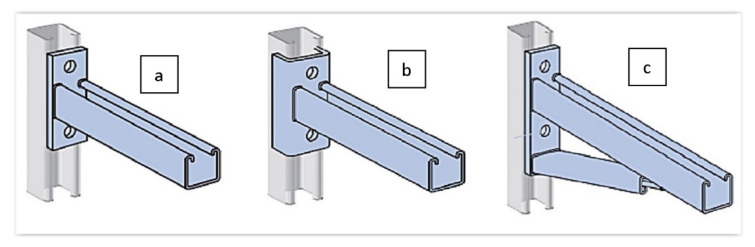
Examples of construction solutions of cantilever brackets designed based on more than one component: (**a**) one profile based design, (**b**,**c**) two or more profiles based designs.

Channel support system struts made based on U-profiles with a channel enable the very flexible design of various systems. The most common solution is cantilever brackets (Figure 1), according to accepted division [3]. They give designers a large field of tactics when designing various assembly systems through a channel. Suppose that one needs to be attached to the wall, ceiling, or flat surface in a traditional system. In that case, the bracket (console) must be equipped with suitable fastening elements, usually welded, and less frequently connected to the frame to attach the bracket to the selected surface.

**Figure 2:**
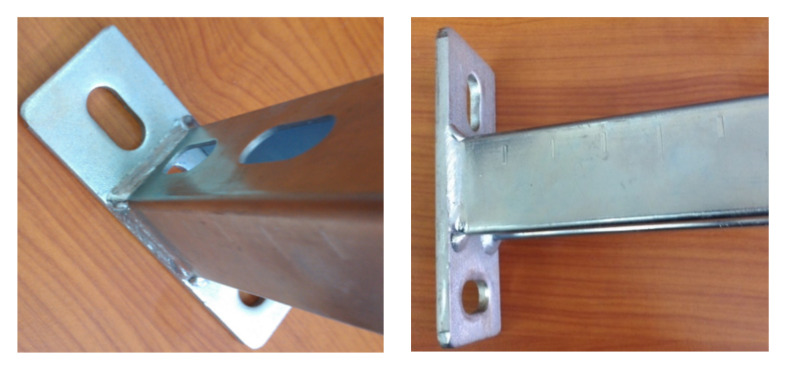
Example of a welded console—a close look at the weld zone after regalvanizing; standard profile of 40 mm × 40 mm cross-section dimensions.

**Figure 3:**
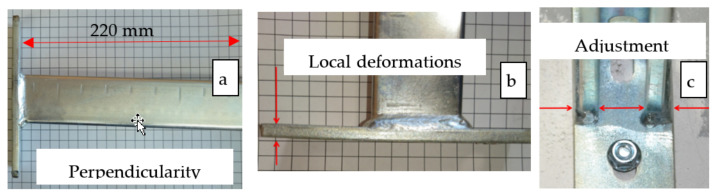
Welded strut design defects: loss of perpendicularity (**a**), deformation of the fastening part (**b**), and defect of the adjustment of the fastening position relative to the support part (**c**). Standard profile of 40 mm × 40 mm cross-section dimensions.

Several construction-solution consoles are used on the market. One group is based on the construction made from one profile section, usually combined with fixing components (Figure 1a). As part of the second group, it is possible to distinguish structures from two or more areas of profiles, respectively, combined to obtain the required formula (Figure 1b,c). In one embodiment, the profile is a carrier element. Another type is a fixing element of the structure, usually using a channel. In the case of consoles responsible for the attachment of other structural elements, the cropped section of the steel profile in the previous solution is combined in the welding process with the steel web stretch that forms the console basis. Then, the incidental resulting from this is subjected to a galvanizing process (Figure 2) to protect the weld surface from corrosion. The method implemented in this way has limited automation capabilities. It is time-consuming and energy-intensive. Due to environmental protection requirements, regalvanizing often forces the manufacturer to seek subcontractors. Welding of profiles made of galvanized sheets causes difficulties with a final component’s high quality (Figure 2). In addition, the product itself is not without defects. As shown in Figure 3, the welded consoles have various shape errors, such as nonperpendicularity of the support element to the fastening element (Figure 3a), local deformations of the fastening part (Figure 3b), and shift errors (Figure 3c).

The numerical analysis permits foreseeing shapes, defects of the products through simulation and efficiently manufacturing the products but also structure behavior under loading. Several works have been done using various methods to analyse either profile roll forming [4] and structure performance [5]. Although studies can be found on the analysis of selected forms of loading profiles [6,7,8,9], the analysis of loading brackets under load is not a subject of extensive research, which is understandable because simple strength analysis is sufficient for industrial applications. On the other hand, the use of specific construction solutions, such as the proposed concept of a lock, requires more accurate analysis. They must be associated even with comparing the stiffness of such a solution to the traditional construction and possible structure optimization, taking into account geometry changes that have an ambiguous impact on the structure. Thin-walled rectangular hollow section (RHS) struts have been confirmed to be vulnerable to various modes of interactions, and have shown sensitivity to different imperfections [10]. Due to their flexibility, most steel thin-walled components are designed to have an ultimate state in the elastic-plastic range [11]. Experimental studies confirmed these observations [12,13]. Since, depending on the load conditions, thin-walled structures susceptible to instability of global buckling as well as to localized deformation [14,15,16,17]. It is especially crucial for a structure like a console, in which the stress is localized around joining/assembled zone.

Taking up the challenge of replacing the existing time- and energy-consuming solution required finding a nontrivial design solution and implementing, in this solution, a series of numerical experiments and laboratory tests have been carried out. For the innovative design solution proposed, in which the welding process was replaced with a mechanical lock, it became essential to find the geometry of this connection. The essence of the solution sought was to develop a lock pattern that would not weaken the structure. This meant that the lock and its immediate surroundings could not be weaker than the rest of the design; i.e., the console’s degradation process should start in other areas further away. Geometrical and technological conditions limited the scope of possible solutions. The geometry of the connection should be as simple as possible and easy to make, and with the necessary accuracy.

### Proposed Construction and Technological Solution

Some solutions were analyzed in the initial phase of the new product. Ultimately, economic conditions and constraints related to the implementation of the mechanical joining process caused the selection of a solution with a specially punched lock. Given the indicated restrictions, modifications of the technological process were proposed based on the production of consoles only on metal-forming processing technology. The new approach eliminated the most significant drawbacks of technology previously used: the difficulty in using full automation, relatively long-term product manufacturing, and the appearance of the earlier-mentioned product defects. An important added value was a significant reduction in the harmful effects on the environment by eliminating welding and galvanizing processes.

**Figure 4:**
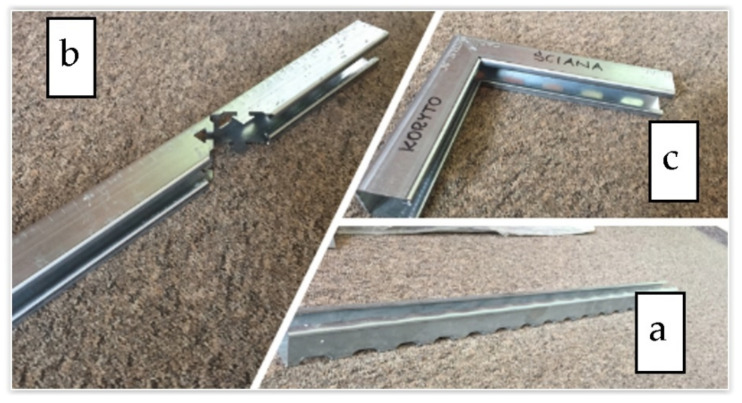
The profile section obtained on the roll-forming line (**a**); a profile section after a laser cutting of a zipper/lock (**b**); and a console with a lock after assembling (**c**). Standard profile of 40 × 40 mm cross-section dimensions.

The technology’s overall concept, which was to replace the traditional welded strut, involved using a particular punched lock, and was based on the possibilities of the current production line. As part of the proposed solution, the console was manufactured from a single fragment of the steel profile, Figure 4a. During the production, the outline of the lock in the profile walls was laser-cut (Figure 4b). The cut-off profile was bent at a straight angle so that the support part and the console fixing part were shaped simultaneously (Figure 4c). Finally, after folding, elements stabilized each other by their interconnection. Such a concept also provided one more option: freedom to select the length of the arms depending on the specific requirements of the constructor or the conditions of the recipient.

**Figure 5:**
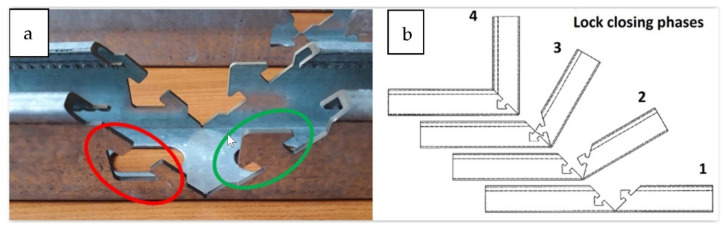
The concept of the console bending process with a lock: (**a**) the laser cut lock outline; (**b**) console assembly process: 1–4 subsequent bending stages.

On the assembly stand, the profile element with the lock cutout shape, shown in Figure 5a, was stabilized in the position shown in Figure 5b (phase 1). Then the console arm was bent upward (phase 2) while the areas (indicated in Figure 5a) were deformed at the elastic limit on both sides of the profile. The red zone, squeezed by an external actuator, was distorted toward the inside of the profile. The green zone was deformed outside the profile by the insert pin. The degree of elastic deformation of the lock elements allowed the lock elements to pass and overlap in phase 3. Then, the expansion pin was removed. The locking elements slid one after another when it was closed. In phase 4, the lock closed when the cut details “clicked” into place, which required high stability and accuracy in performing individual operations.

The proposed solution concerning the primary welded console had many advantages, including, among others:

- Eliminating the heat source avoids potential deformation of the fastening part;
- Bending the profile guaranteed the perpendicularity of the console elements; i.e., the bracket and the fastening portion, and also eliminated the possibility of switching these parts relative to each other;
- Execution of the console entirely from the profile caused the bracket and fixing part to be the same width, and thus allowed the sliding of two consoles toward each other and shared assembly (the situation occurred when more outstanding durability was required);
- The console can be produced from any material from which the profile was made (in the case of a welded console, the material change entails the need to select new parameters—or even welding methods).

Undoubted benefits resulting from the use of a new console implementation technology are associated with manufacturing (better possibilities of production automation) and pollution reduction toward green technology (higher level of reduction from production components that are difficult to recycle). However, it must be verified whether such a concept did not differ significantly from the traditionally used welded consoles when it came to the strength of the structure, which was the main purpose of using such components.

**Figure 6:**
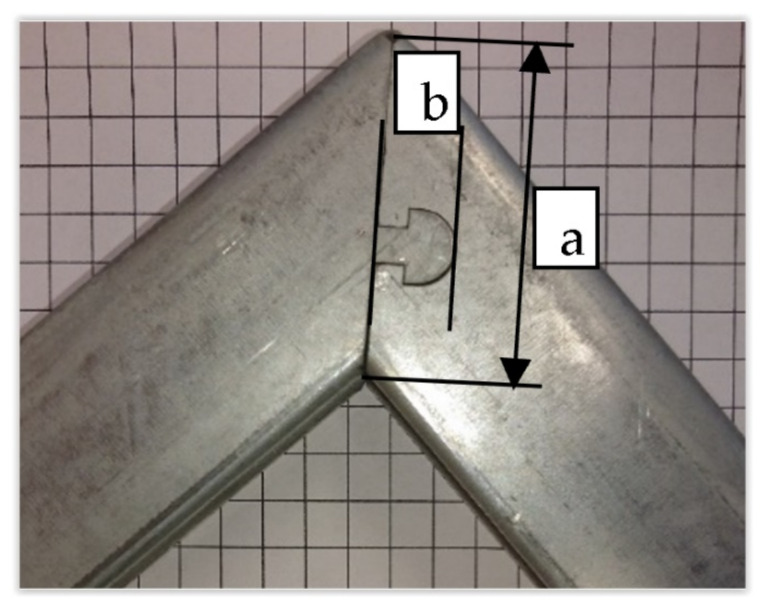
Limitations of the positioning of the lock: (**a**) chord, (**b**) lock height.

The introduction to this research was to propose the concept of a lock geometry. Then, a numerical analysis of the presented cases was carried out in terms of the strength of the structure as analyzed by construction deflection, compared to the traditionally used welded solutions. The newly proposed solution included the execution of two procedures: bending the profile at a suitable angle and alleviating two parts of the console relative to each other using a specially shaped lock. It was assumed that the shape of the lock’s fastener might affect its difficulty, subsequent assembly, and the strength of such a solution. The preliminary work focused on analyzing possible variants of the lock cutting. The first problem proved to be the determination of the dimensions of the lock shape due to technological and economic capabilities. The primary limitation on the punched outline of the lock (dimensions and position of the rounded and triangle clip) was the possibility of adequate placement on the side of the profile. Tests showed that due to the technology of the lock outline notch, depending on the width of the profile wall, the height of the cutout (Figure 6) could not exceed half of the string to meet the tasks of adequate fastening and executability.

**Figure 7:**
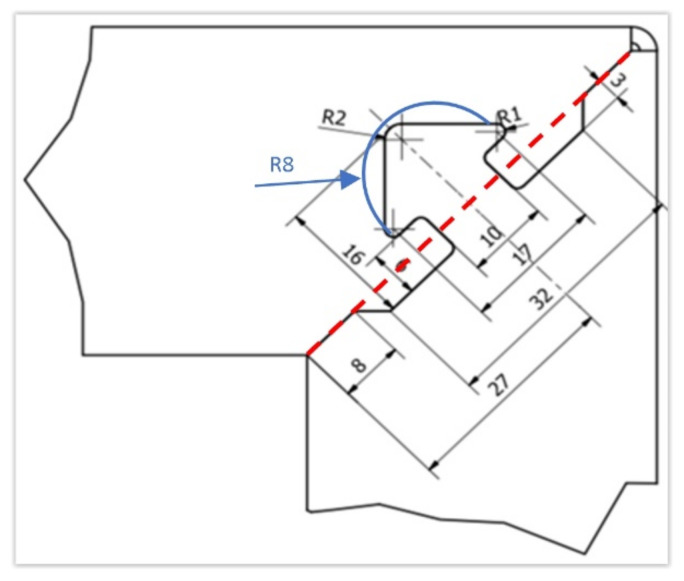
Basic concepts of lock dimensions—essential dimensions of the lock and specific dimensions of different lock tips.

Two basic shapes of the lock, semicircular (rounded clip) and triangular (arrow clip), were used (Figure 7), with three variants: basic cutting line, cutting line with step, and opposite direction of the lock tip. Details of the shape/dimensions (undercutting, rays, etc.) were selected based on the recommendations of technologists that took into account the precision of the cutting process.

**Figure 8:**
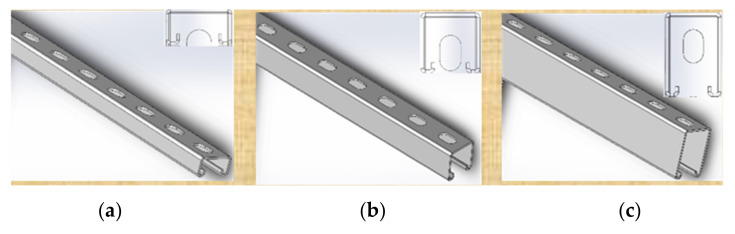
Profile cross-section types: (**a**) 40 mm × 20 mm; (**b**) 40 mm × 40 mm; (**c**)—40 mm × 60 mm.

A strut based on one type of profile in three cross-sections types (40 mm × 60 mm, 40 mm × 40 mm, and 40 mm × 20 mm, with a 2.0 mm thickness) for the same geometry were considered. Figure 8 presents profiles with corresponding cross-sections. Numerical analysis were carried out for all three profile cross-sections, while validation experiments were conducted for the 40 mm × 40 mm profile only.

## 2. Materials and Methods

### 2.1. Numerical Modeling

The numerical model assumed that the load was static, while plastic deformation of the material was permissible. Therefore, nonlinear static analysis was selected. The source of nonlinearity was the description of the elastic-plastic material model and contact phenomena on the surface contact in the console zone, as well as between the pin forcing the deformation and the console (Figure 8). An isotropic material model with a description of von Mises plasticity and kinematic hardening was assumed. Material anisotropy was omitted due to a small impact on the deformation of the console under load. A Hollomon equation (σ_pl_ = C ε^n^) was used to describe the stress–strain relationship. In order to determine material parameters C and n, standard stretch attempts were made. An MTS Bionix machine was used for testing. The test steel was DX51 (1.0226 according to EN10027) [18,19,20]. The material data list is shown in Table 1.

Contact phenomena were modeled using friction according to the Coulomb model. Because mutual movement of contact zones did not have a significant impact on the final results, a detailed determination of the friction coefficient was omitted. A friction coefficient µ = 0.1 was assumed based on a comparison of the numerical and experimental results, as described in Section 2.2.

**Figure 9:**
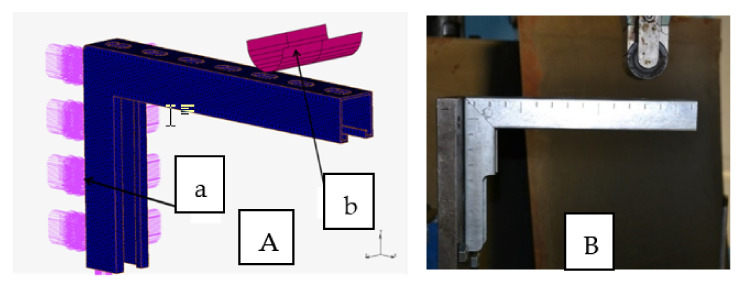
Concept of the console load: (**A**) numerical model (a—fastening, b—loading pin); (**B**) method of experimental implementation of the numerical research.

From the point of view of the accepted boundary conditions, it was crucial to properly attach the console during the load testing. The applied load system is shown in Figure 9A. The boundary conditions applied in the numeric model in the form of degrees of freedom applied around the holes on the side of the console to reflect the method of fastening used in the laboratory tests of the console load are also shown. Because the console prototype tests were rigidly loaded to the press beam with a rolling pin, the shape of the shaft was taken into account, and a rigid model of this element was adopted. The exemplary implementation of research on the test bench is shown in Figure 9B.

### 2.2. Optimization and Validation of the Numeric Model

In the initial analysis phase, a standard procedure considered optimization and validation/verification of a virtual numerical model [21]. Numerical modeling aimed at checking the proposed new console structure solution took place in three stages:

- Optimization of the numerical models for applied finite elements—a type of 3D elements and mesh density; it was assumed that the verifying criterion would be the maximum equivalent stress observed in the area of the lock, around which stress concentration was expected;
- Validation of the adopted material models for the console and boundary conditions based on the laboratory trials to load selected consoles samples; as a criterion, the force course was adopted;
- Analysis of the new structure.

In the initial phase of the modeling, several series of simulations were carried out to check the legitimacy of the 3D model. In justified cases, the 2D model with shell elements (2D shell) was more convenient due to a much simpler geometric model required to prepare the finite element mesh. In addition, the mesh itself was more convenient to construct. For very extensive models, the use of the 2D model was more effective, due to often clearly shorter calculation times and, therefore, the ability to analyze more cases in a shorter time. Basing on earlier experiences and a literature review concerning numerical modeling of steel profile analysis, a 3D model was assumed, and one geometry of the console carried out on a technological line was selected to verify the simulations.

On the basis of the initial analysis, the TETRA elements were used in models assuming that they would offer more accuracy, reflecting the geometric complexity around the developed locking lines of the locks. First- and second-order elements were used, characterized by four (TETRA4) and eight nodes (TETRA8). After performing the load simulation for five mesh densities in two variants of finite elements, the results were analyzed for the maximum values of the recorded equivalent stresses (observed) under load (Table 2). In the case of numerical modeling performed using the software based on the finite element method, it was assumed that smaller elements made the simulation results more accurate. A disadvantage of the decrease in mesh density was obvious: smaller finite elements in the model could significantly extend the calculation times, which increased with the increasing number of elements. The same phenomenon was observed when higher-order finite elements were implemented instead of lower-order finite elements: better accuracy accompanied the increase in calculation time. Optimization of the 3D element type indicated that the model with TETRA4 elements exhibited comparable values of the maximum equivalent stresses to those of TETRA8 observed around the lock. Still, it was more effective from the point of view of the calculation time, which was approximately 20–30% shorter than when using TETRA8 elements.

**Figure 10:**
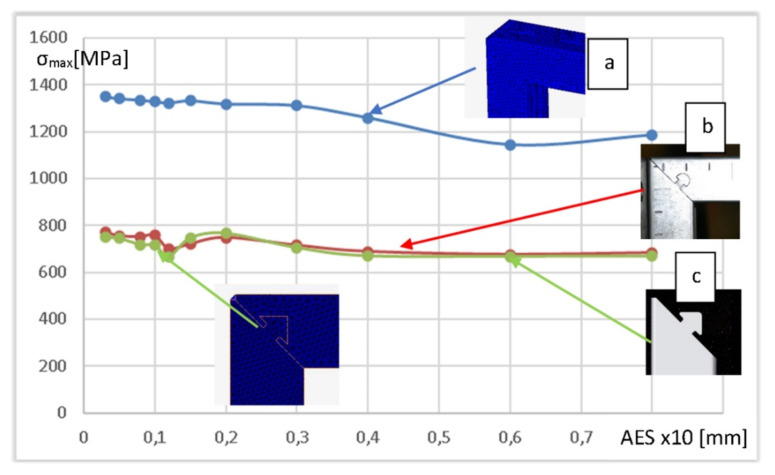
Selected results of the optimization procedure for three numerical models with a profile of 40 mm × 40 mm: console without lock—virtual strut (**a**); console with semicircular shape lock (**b**); console with arrow lock (**c**).

The standard optimization procedure was required to be applied to ensure accuracy and efficiency in the solution. It aimed to analyze the initial mesh size (number of elements) that influenced the results (selected physical quantity). A set of simulations were conducted with different finite element geometry (density/size) parameters to achieve that goal. It was reasonable to focus on model areas at which the concentration of stresses was expected, and to refine the mesh to assist in the reduction in errors and result in a better approximation to the mathematical model. The simulations repeatedly changed the average distance between the mesh element nodes (average element size—AES). When increasing this value, the density of the nodes would decrease, but the quality of the mesh quality may have suffered as a result. If we decreased this value, the mesh quality would improve, but the number of elements could grow dramatically, causing a rapid computation time. The characteristics of maximum equivalent von Mises stress peaks vs. AES, presented in Figure 10, generated based on three console cases (with a semicircular lock, with an arrow lock, and an ideal strut without a lock) with a profile of 40 mm × 40 mm suggested that it stabilized around 2 mm AES. However, a curious drop in the stress value of 1.2 mm was observed. This probably was related to the adverse effect of geometry on the local mesh layout. Confirmation of such a hypothesis was visible to stabilize solutions for subsequent, smaller AES values. Therefore, the next step was to use the results computed with AES equal to 1 mm as optimal and as a starting point for further analysis and comparisons.

The numerical model optimization indicated that the model with TETRA4 elements while maintaining a 1 mm edge of the finite element was optimal for accuracy and calculation times. The graph in Figure 10 indicates an apparent stabilization of the results when there was a further reduction in finite elements: for four sequential mesh densities, there was an increase in the level of maximum stresses below 2% (for lock b) and below 4% (for lock c).

**Figure 11:**
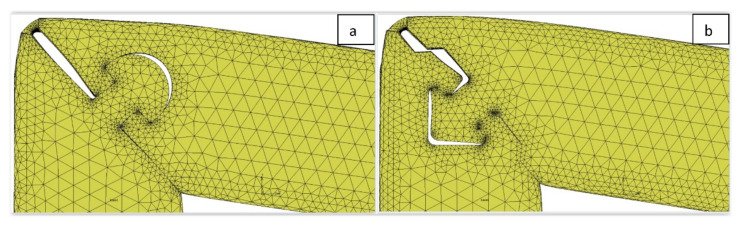
Examples of mesh modifications around the semicircular (**a**) and arrow lock areas (**b**).

The optimization of the numerical model was intended to increase the overall accuracy of the results. In a situation in which a distinct concentration of stresses/strains is expected, as is in the case of the lock mechanism used, the optimization of the shape of the mesh in this area is extremely important. Based on the analysis of stress distribution around the zone of the lock, modification/concentration of the mesh was proposed, as shown for two cases in Figure 11. This was aimed at improving the accuracy of determining the quantities of derivatives in those areas that could more precisely follow local weakening of the structure.

**Figure 12:**
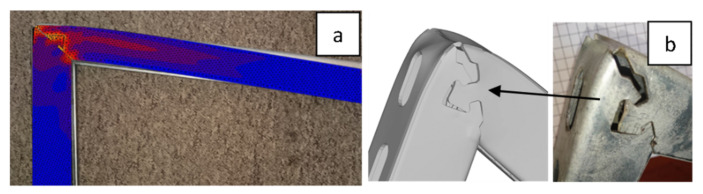
Comparison of simulation results and laboratory test loads of the selected prototype of the console: console deflection (**a**); deformation of the lock (**b**).

The numerical model was validated by comparing the forces and shapes between the selected results with a triangular lock and a prototype console under a given load. Figure 12 shows the differences in the form obtained in the numerical modeling and the static attempt to load the prototype structure. The deflection of the console (Figure 12a) and the shape of a deformed lock (Figure 12b) were compared. The differences in the set forces for the given displacement were less than 1%.

**Figure 13:**
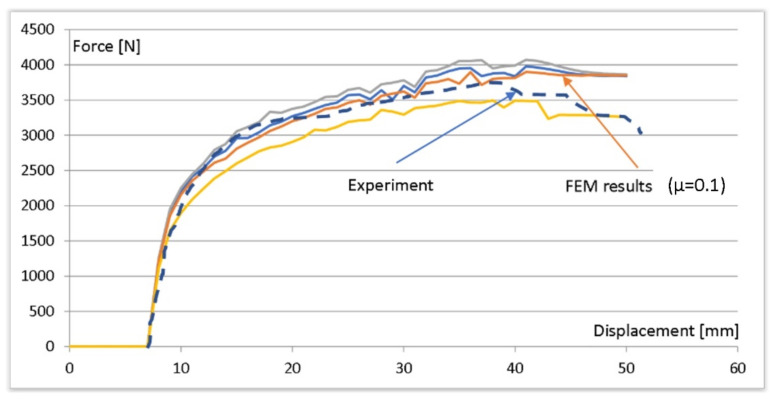
Comparison of force during loading for the friction-coefficient establishment (console 40 mm × 40 mm; arrow lock).

For establishment of a general friction coefficient, the force characteristics calculated for different friction coefficients were compared with those recorded during attempts to load the console on a specially designed stand. The obtained results were used to determine which best reflected the real conditions of the friction coefficient, which resulted in a good adjustment of the force obtained from the numerical simulation to the data obtained experimentally. Figure 13 presents a comparison of selected results for the console with the arrow lock. The registered course of force in the laboratory conditions is marked with interrupted line. It was considered that the most accurate match of force was obtained for a coefficient of friction µ = 0.1.

## 3. Results

**Figure 14:**
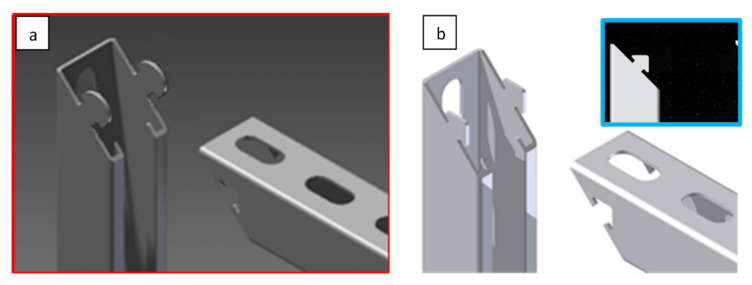
The 3D models of two different lock variants: semicircular (**a**); triangular (arrow) (**b**).

**Figure 15:**
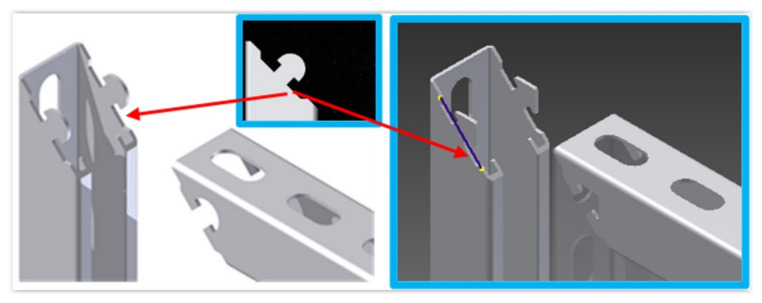
The 3D models of the consoles with a modified contact line (with a dash/shelf) for two lock cases: semirounded and triangular.

The console modeling focused on assessing the performance of the strut, particularly the load capacity, and developed numerical models aimed at the analysis of the impact of the selected geometry of the lock. As a reference console (benchmark), a virtual solution without a lock was used for the strength analysis, as shown in Figure 10a. The analysis of such a case allowed us to reveal particularly burdened areas, which in turn granted the confirmation of the assumptions for the design of the lock shapes. The solution without a lock was ideal; that is, it had the highest possible strength, which could be used comparatively to assess the proposed solutions. In further work, the accepted solutions (the semicircular and triangular locks shown in Figure 14a,b) were modified to analyze the additional stiffening of the structure. The final attempts used two variants (semicircular and triangular) with a modified contact line, the so-called shelf/dash. The adopted solutions are shown in Figure 15.

The numerical analysis consisted of two types of attempts: analysis of the deflection range with a fixed load, and testing of the deflection with the assumed maximum level of equivalent stress initiating material plasticity. 

**Figure 16:**
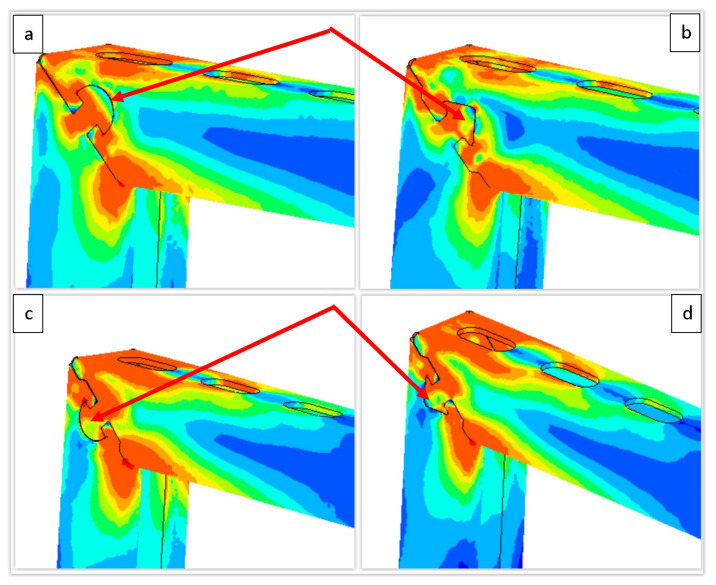
Exemplary FEM results showing distributions of equivalent stresses around the lock: semicircular (**a**) and an arrow (**b**) pointing down (**c**) and up (**d**), respectively. Standard profile of 40 mm × 40 mm cross-section dimensions.

Numerical experiments were aimed at an analysis of stress fields around the locks caused by the applied load. The analyzed stress distribution suggested a similar level of maximum equivalent stresses for the cases studied, Figure 16. However, the most subtle nature of the development of stress fields was obtained for the semicircular lock, Figure 16a. This would suggest a more stable behavior of the lock, and potentially subsequent occurrence of damage effects. This was confirmed when comparing the forces for selected cases. In the elastic deformation range, the semicircular lock showed a more significant impact on the improvement of the observed deflection of the console. 

The observations of the simulation results in the form of distribution of equivalent stresses also indicated that the use of “shelves” in the lock resulted in a favorable concentration of stress, leading to better system locking. Still, simulations did not confirm the apparent improvement of the structure’s deflection. This meant that the assumption of more effective lock blocking during loading was correct. However, the waveforms calculated for the loaded systems showed that this did not significantly increase the strength within the limitations assumed by designers. The differences demonstrated concerned only the nature of deformation of the lock during its load.

**Figure 17:**
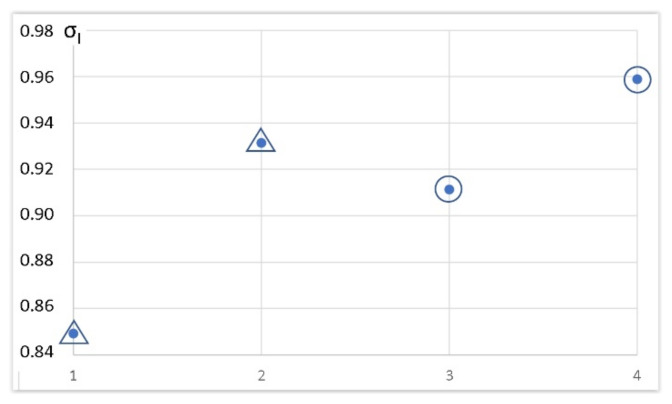
The calculated indicator σ_I_ for consoles with selected locks: 1—arrow lock; 2—arrow lock with dash; 3—semicircular lock; 4—semicircular lock with dash.

Figure 17 shows the determined values of the yield stress indicator σ_I_ for arrow and semicircular locks (with and without a dash), indicating the ratio of yield stress to the material used on the consoles for the calculated equivalent stress (1):

σ_I_ = σ_p0_/σ_eq_ (1)

The homogeneity of the stress distribution around the lock was essential to its load capacity. Therefore, if the indicator σ_I_ increased, the danger of the appearance of plastic deformations was reduced. Again, the console with a semicircular lock showed a somewhat higher resistance, this time to the formation of local plastic deformations around the lock.

Based on the numerical modeling results, the validation was carried out using experimental testing prototypes of selected solutions/concepts. The maximum displacement of the loading pin of 100 mm was applied to obtain the plastic deformation range for the consoles chosen with the adopted locking variants. Such studies were conducted on 40 mm × 40 mm cross-section profiles. Due to the statistical evaluation for each type, seven repetitions were performed. The method to fix the console on the position was prepared according to the diagram in Figure 9a. 

**Figure 18:**
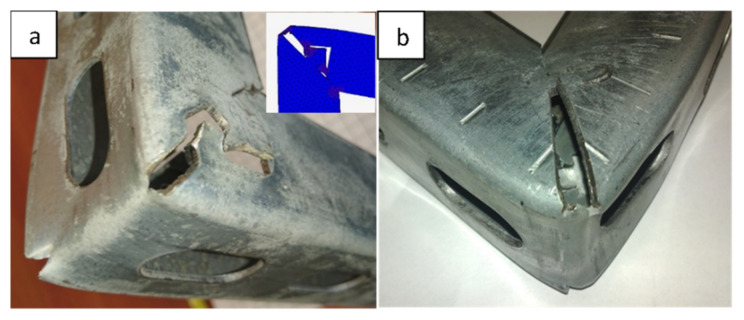
Consoles after testing—no catastrophic destruction regardless of the shape of a lock; (**a**)—triangular lock with shelf; (**b**)—semicircular lock without a ledge.

**Figure 19:**
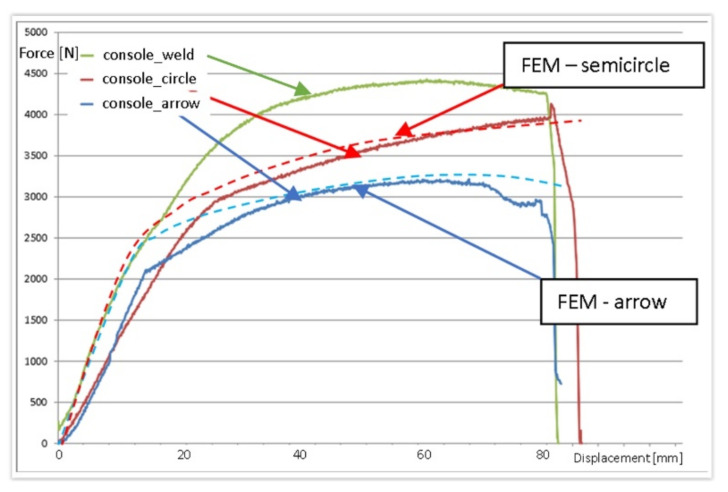
Comparison of forces obtained during loading of the console for selected cases of experimental tests and FEM simulations with a profile cross-section of 40 mm × 40 mm: semicircular lock; arrow lock; welded console.

Research conducted at an experimental station showed that optimized numerical models with sufficient accuracy reflected the preservation of the designed console in the context of the structural deformation (Figure 18) and the course of loading force (Figure 19). Figure 19 shows selected recorded loading results from a 40 mm × 40 mm profile console with a section of semicircular and arrow shape locks with a flat contact edge compared to the welded console. A slightly more accurate mapping was obtained for the console with a semicircular lock. Almost the same characteristics were observed for the 40 mm × 60 mm profile, but the differences between locks and welded consoles were minor.

The results obtained indicated that the analysis of derivatives, such as stresses, could be carried out in a relevant manner. It is worth noting that within the accepted scope of the console’s burdens, the consoles with a lock was not destroyed in a catastrophic way. After bending (loss of load capacity), both parts, fixing and supporting, remained united with each other, as shown in Figure 18. This was vital due to the safety of using new consoles. There are several documented cases of welded consoles in which the breaking of the weld and separation of the fastening part from the support were observed in production practice.

## 4. Discussion

The proposed solution system applied to the construction of a support section in one profile section using the punching process to obtain a unique lock without performing any mechanical or welded joints. The endurance of such a solution and the impact of lock geometrical parameters were a matter to clarify the explanation. The analyses were concentrated on local stresses and global console deflection. 

The study of the cases examined dealt with a deflection range with a fixed load and the testing of deflection with the assumed maximum level of equivalent stress initiating material plasticity. It was found that in both cases, taking the most negligible deflection under the load and the lowest level of equivalent stresses causing material plasticity around the zipper area with increasing deflection, the most preferred solution was the use of a semicircular lock. Similar dependencies were obtained independently of the boundary conditions assumed. The safety coefficient, for which the limited value of equivalent stresses was considered, differed between these cases from 15% to 50%. Comparable values were obtained for a double (inverted) triangular lock.

From the analysis of force flow during the static loading, it followed that the lock-based consoles showed somewhat greater susceptibility to loads—they showed deformations with a smaller level of force (Figure 19). However, these differences were only in the field of emerging plastic deformations. Within the elastic range, these differences were relatively more minor. Still, in experimental studies, destructive deformations of the locking consoles with locks were not observed in the examined force limits. On the contrary, single cases in industrial tests of welded consoles were signaled. The conducted research suggested that these cases could have resulted from welded connections, which generally decrease durability. However, if so, this would mean that the solutions with the lock were less vulnerable to manufacturing technology errors than the welded constructions. Experimental tests showed that loads of consoles with locks within the adopted range did not cause destruction (Figure 17). In the lock zones, the consoles were significantly deformed, but were still working, without catastrophic effects. 

**Figure 20:**
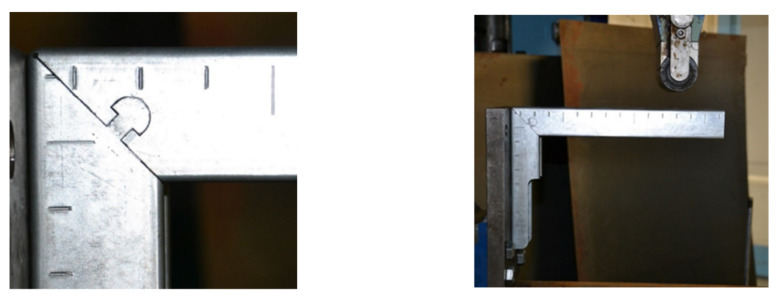
Correctly made console with a semicircular lock in close-up; console before loading on the experimental stand (40 mm × 40 mm profile).

The best results from both points of view, deflection of the console and local plastic deformation, were obtained for the console with a semicircular lock shape, as shown in Figure 20.

The comparative analysis carried out showed satisfactory compliance of the results obtained. The introduced construction changes allow the automation of console production and implementation in one technological string. Improvement of the initial lock with the “dash” caused a smoother stress distribution around the lock, leading to a better locking of the system. We could conclude that the assumptions were correct, but both simulations and experimental studies did not confirm the evident improvement in the deflection level of the strut structure. The differences only concerned the nature of deformation during loading. Since the execution of such a lock requires good accuracy of the cutting planes, this did not seem to be a meaningful solution.

**Figure 21:**
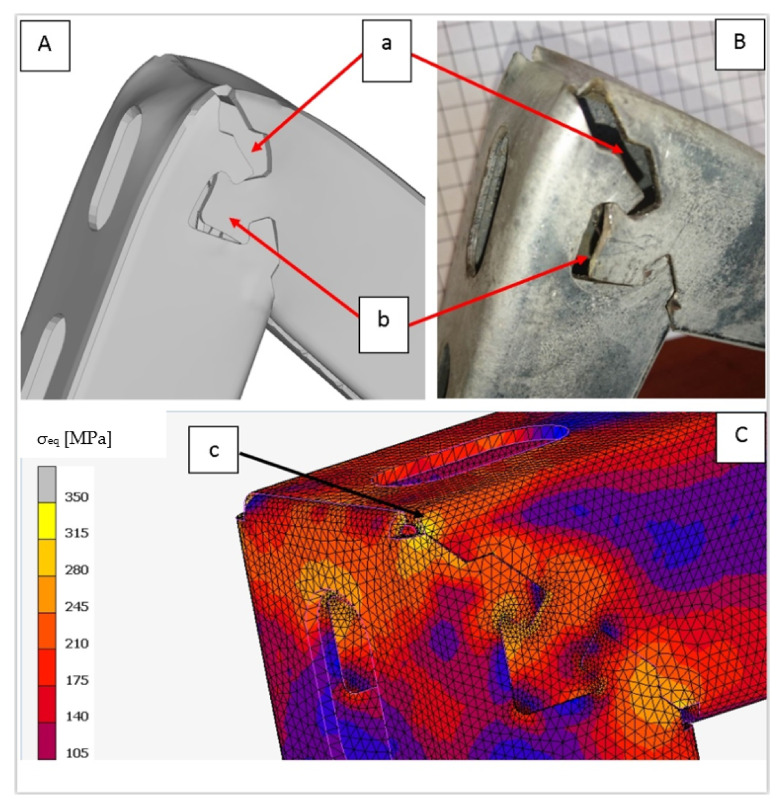
The degradation of the console top edge: results of numerical simulation (**A**) and stand tests (**B**); stress distribution at stage at which degradation start; degradation zone—a, lock deformation—b; initial degradation zone—c (**C**).

Due to the variable geometry of the consoles, including the height of the bracket, as shown in Figure 8, each size of the console had a different area, defined by dimensions a and b shown in Figure 6, in which the lock had to fit. Variable geometry made it possible to design dedicated solutions, giving better results. However, based on the preliminary assumptions made regarding the ease of constructing the lock, including the organization of production, speed of execution, and shape accuracy, a universal solution was adopted for the entire series. The second aspect was verified for the solution adopted in this way; i.e., the strength of the locked zone and its surroundings. The developed solution fulfilled the assumption that the console would be damaged outside the locked area. The results of numerical modeling shown in Figure 21A and the corresponding results of the stand tests (Figure 21B) indicated the destruction of the upper edge of the console. Degradation occurred when the allowable tensile stress was exceeded.

## 5. Conclusions

The effects of material lock system variations were analyzed by developing FEM models and validation of selected prototypes. Various types of consoles/struts were validated against existing test data. The analysis of the geometry of the lock and the impact on console performance, including its load capacity, were carried out. Designed solutions were constructed in MSC Marc, an environment dedicated to advanced modeling and numerical analysis. A numerical study corresponding to the operating conditions and destructive loads was performed. It was assumed that three-dimensional FEM models were performed statically and isothermal. Numerical models were optimized for the amount and type of finite elements used. To achieve this goal, a simplified approach was introduced to determine the strength of the initial geometry, including residual stresses. The following conclusions were drawn from the numerical and laboratory experiments:

- The application of the locks did not distinctly lower the bearing capacity of the console compared to traditional (welded) solutions;
- Lock geometry modification in the form of the “dash/shelf” improved the stability of the structure, although without a significant, worthy console deflection decrease;
- Evaluation of the numerical analysis results for stress fields around the locks suggested that the most effective solution was the use of a semicircular lock, and the difference between the arrow and semicircular locks observed in force characteristics during loading was between 10–15% in favor of the latter;
- Experimental validation for the 40 mm × 40 mm profile confirmed the main conclusions drawn from the numerical analysis, that the semicircular lock performed the best from the point of view of the general console’s strength under the assumed loading scheme; the construction began to yield somewhat later, and deformed under higher force.

In future work, during experiments, the new concept of lock mechanism may be sensitive to geometry imperfections; in other words, the cutting technology accuracy. Therefore, it seems logical to check to what extent inaccuracies in the cutout of the lock will affect its quality (strength/durability). Thus, there is a need to analyze selected scenarios of lock imperfections for each type of console while taking into account the influence of inaccuracy on the degree of stress concentration in the corners of the upper edge, which are the points of crack initiation.

## Figures and Tables

**Table 1:** Material model data for numerical analysis.

Young Modulus—E	Poisson’s Ratio—ν	Yeld Stress—R_e_	C	n
210,000 (MPa)	0.3	310 (MPa)	560 (MPa)	0.194

**Table 2:** Sample values of maximum equivalent stress for selected 3D elements.

Element Size [mm]	σ_eq max_ [MPa]	
	TETRA 4	TETRA 8
1	615	621
2	612	619
4	698	616
6	589	595
8	568	577

## Data Availability

Not applicable.
